# Unraveling Desmin’s Head Domain Structure and Function

**DOI:** 10.3390/cells13070603

**Published:** 2024-03-29

**Authors:** Dimitrios Vlachakis, Konstantinos Tsilafakis, Ioanna Kostavasili, Sophia Kossida, Manolis Mavroidis

**Affiliations:** 1 Biotechnology Department, Agricultural University of Athens, 11855 Athens, Greece; dimvl@aua.gr; 2 Center of Basic Research, Biomedical Research Foundation, Academy of Athens, 4 Soranou Ephesiou, 11527 Athens, Greece; constantints@hotmail.com (K.T.); ikostavasili@bioacademy.gr (I.K.); 3 Biochemistry & Biotechnology Department, University of Thessaly, 41500 Larisa, Greece; 4 IMGT, The International ImMunoGeneTics Information System, National Center for Scientific Research (CNRS), Institute of Human Genetics (IGH), University of Montpellier (UM), 34090 Montpellier, France; sophia.kossida@igh.cnrs.fr

**Keywords:** desmin, NDUFS2, saposin D, protein interactions, homology modelling

## Abstract

Understanding the structure and function of intermediate filaments (IFs) is necessary in order to explain why more than 70 related IF genes have evolved in vertebrates while maintaining such dramatically tissue-specific expression. Desmin is a member of the large multigene family of IF proteins and is specifically expressed in myocytes. In an effort to elucidate its muscle-specific behavior, we have used a yeast two-hybrid system in order to identify desmin’s head binding partners. We described a mitochondrial and a lysosomal protein, NADH ubiquinone oxidoreductase core subunit S2 (NDUFS2), and saposin D, respectively, as direct desmin binding partners. In silico analysis indicated that both interactions at the atomic level occur in a very similar way, by the formation of a three-helix bundle with hydrophobic interactions in the interdomain space and hydrogen bonds at R16 and S32 of the desmin head domain. The interactions, confirmed also by GST pull-down assays, indicating the necessity of the desmin head domain and, furthermore, point out its role in function of mitochondria and lysosomes, organelles which are disrupted in myopathies due to desmin head domain mutations.

## 1. Introduction

Desmin is a member of the large multigene family of IF proteins and is specifically expressed in myocytes. Like all IF proteins, desmin (53.5 kDa) exhibits a tripartite structure consisting of an amphipathic central α-helical coiled coil domain (“rod”) flanked by the flexible “head” and “tail” domains. The rod domains are more than 90% sequence identical among different species. Due to their intricate architecture, the 3D structure of IFs has remained elusive. The current structural model of the desmin rod domain is derived from structural analyses of the closely related class III IF protein vimentin [1]. The rod domain consists of two continuous α-helical segments, which are interconnected by a linker polypeptide sequence. Rod domain coil 1 itself comprises two α-helical subdomains, which are tied by a second linker sequence [1,2,3]. The sequence/structure of the rod domain is mainly what distinguishes intermediate filament proteins from other coiled coil-forming proteins and defines a bona fide intermediate filament [4]. In contrast, the “head”- and “tail”-domains are structurally more variable, both in terms of length and primary sequence, between different IF members. Nevertheless, within the head domain, a nonapeptide motif “SSYRRTFGG” at the amino terminus has been discovered to be evolutionarily highly conserved within class III IF proteins as desmin, vimentin and peripherin [5,6].

There is no full 3D structure information for the flanked flexible low-complexity domains. However, in the tail domain of lamin A/C, an Ig-like domain with a β-sheet formation in dimer has been characterized [7,8]. However, recent solid-state NMR spectroscopic studies of neurofilament’s light protein and desmin’s head domain polymers reveal spectral patterns consistent with structural order and β-strand secondary structure [9]. They proposed that head domains, even in the context of assembled IFs, somehow are dynamic and continuously moving in and out of the structurally ordered state [9], so they are accessible for interactions with other proteins and post-translational modifications. Likewise, for head domain self-association, a complete atomic model of vimentin IFs suggest that the protofibrils interact laterally via the tail domains, while the head domains form an amyloid-like fiber consisting of labile cross-β conformations in the lumen of vimentin IFs [10,11]. In addition, in the full-length vimentin dimer, the head domain seems to fold back on coil1A, as SDSL-EPR data indicated an interaction of residue 17 of the head domain and residue 137, located near the C-end of coil1A [12,13].

The variability in the amino terminal head domains and the carboxy terminal tail domains is responsible for the diversity, specificity and regulation of intermediate filaments [14,15]. It has been proposed that the head and tail domains protrude from fully formed IFs, allowing access to other cellular proteins. This raises the possibility that head and tail domains play another role, other than filament assembly and tissue-specific stability, although it has been shown that the “head” domain is required for proper IF assembly [16,17,18]. One could hypothesize that, through evolution, the sequence of the head and tail domains have adapted, by making a particular IF protein well-suited for one cell type but poorly suited for another.

Two phospho-sites (S28 and S32) in the desmin head domain are connected to canine and human heart failure (HF), where the accumulation of mono-phosphorylated cardiac desmin was correlated with cleavage in the head domain, which led to pre-amyloid oligomers (PAOs) formation [19]. Also, the same group, using an in vitro model, confirms GSK3’s involvement in desmin phosphorylation [19]. In fact, this kinase is known to phosphorylate a Ser, three amino acids upstream from another Ser, which has been phosphorylated by another kinase (priming effect) [20]. The pSer32 seems to be the substrate for the nucleation process, leading to cardiac toxic PAOs deposition [21].

These residues are localized in the N-terminal head domain of desmin, a portion of the protein known to be critical for its in vitro susceptibility to phosphorylation and proteolytic cleavage and for its role in the assembly of mature IFs [22,23].

Mutations, such as S13F and R16C in the head domain of desmin, alter its assembly and network-forming properties [24]. The S13F mutation has been correlated with desmin myopathy, heart block [25] and ARVC [26], while R16C has been correlated with restrictive cardiomyopathy and atrioventricular block [27]. All the above point out the importance of the desmin head domain in cardiomyocyte structure and function. In an effort to elucidate the role of desmin’s head domain, we screened a cardiac library using the yeast two-hybrid system, and two new binding partners were identified, namely mitochondrial protein NDUFS2 and lysosomal saposin D.

## 2. Materials and Methods

### 2.1. Yeast Two-Hybrid Library Screening

The Matchmaker GAL-4 two-hybrid system was used as described by the manufacturer (Clontech, Clontech Laboratories, Inc., Palo Alto, CA, USA). A cDNA fragment, encoding the NH_2_ terminus of mouse desmin (first 1–320 nucleotides), was inserted downstream of the GAL4-DNAbinding domain in the pGBKT7 bait vector. A yeast two-hybrid cDNA library (Clontech) derived from human heart muscle was screened for interacting proteins as described by the distributor. For interaction, positive clones were selected in a selection medium (SD/-Trp/-Leu,/-Ade/-His +X-α-galactosidase plates), as we have previously described [28]. 

### 2.2. Generation and Purification of Glutathione S-Transferase (GST) Fusion Proteins

The desmin, the NDUFS2 or the prosaposin cDNA fragment was inserted into the pGEX-5x-1 plasmid vector (Amersham Biosciences, Piscataway, NJ, USA) at the EcoRI/XhoI sites, to generate GST fusion proteins. The recombinant polypeptides were expressed into BL21 bacteria by induction with 0.1 mM isopropyl β-D-1-thiogalactopyranoside for 4 h. The bacteria were harvested by low-speed centrifugation, resuspended in lysis buffer 1:20 mM Tris, pH 8.5, 5 mM EDTA, 5 mM DTT, 0.2% (*v*/*v*) Triton X-100 with protease inhibitors (#P8340, Sigma, Darmstadt, Germany) plus 2 mM phenylmethylsulfonyl fluoride). Lysozyme was added to 1 mg/mL and incubated for 1 h at 4 °C. The suspension was centrifuged at 11,000 rpm for 20 min. The inclusion bodies, which contained the GST–desmin, GST–NDUFS2 or the GST–prosaposin fusion proteins, were dissolved in lysis buffer 2, containing 6 M urea, 20 mM Tris, pH 8.5, 5 mM EDTA, 5 mM DTT and 5% (*v*/*v*) glycerol and dialyzed against buffer 3:100 mM NaCl, 20 mM Tris, pH 8.5, 0.5% (*v*/*v*) Tween 20, 1 mM EDTA, 1 mM DTT, 5% (*v*/*v*) glycerol.

### 2.3. Preparation of Desmin-Enriched Cardiac Extracts

Cardiac tissue was homogenized in PBS, 0.6 M KCl, 1% (*v*/*v*) Triton X-100 with protease inhibitors, and the homogenate was centrifuged for 10 min at 3000× *g*. The pellet was resuspended in gel filtration buffer (PBS, 6 M urea, 1 mM EDTA and 2 mM DTT with protease inhibitors) and was further purified with gel filtration column chromatography using the Shodex KW-804 column (Thomson Instruments, Clear Brook, VA, USA) connected to an AKTAP900 purifier (Amersham Biosciences). Fractions enriched in desmin were chosen by SDS-PAGE and Western blot analysis as previously described [29].

### 2.4. Preparation of Total Protein Cardiac Extracts

Mouse cardiac tissue was homogenized in an extraction buffer containing 10 mM Tris, pH 8.5, 0.01% (*v*/*v*) SDS, 20 mM NaCl, 0.01% (*v*/*v*) Nonidet P-40, 0.01% (*v*/*v*) DOC, 5 mM EDTA, 2 mM DTT, and protease inhibitors. The homogenate was centrifuged for 10 min at 3000× *g*, and the supernatant was used as a source for NDUFS2 in GST pull-down assays.

### 2.5. Desmin Head Deletion Protein Preparation

#### Plasmid Vector Construction

The wild type desmin and DesminΔ1-48 cDNAs were subcloned into the pRc/CMV plasmid (Invitrogen Corporation, San Diego, CA, USA), under the control of a CMV promoter. For the generation of DesminΔ1-48 (missing the first 48 aa), the primer “TGTGCAAGCTTGCCACCATGACATCCCGCGTGTAC”, which encodes the desmin protein starting at amino acid 49, methionine (M T S R V Y…) and flanks a HindIII site and a Kozack sequence at the 5 end, was used.

### 2.6. Cell Culture Transfection and Total Protein Extracts Isolation

The COS-7 cell lines were transfected with the above wild type desmin and desminΔ1-48 cDNAs, as follows: transfections were carried out using 1 μL of lipofectamine 2000 (Invitrogen) in 400 μL of Opti-MEM, mixed with the transfection DNA sample and added to the cells after replacing the media with 1 ml of minimal essential medium (MEM). After 5–6 h, the medium was replaced with MEM supplemented with 10% fetal bovine serum (FBS) and the cells were grown at 37 °C in an air—5% CO_2_ incubator.

Total protein extracts from transfected cells (with the indicated plasmids) were isolated from a confluent 10 cm^2^ culture as follows: Cells were homogenized in a buffer containing: 20 mM Tris pH 6.8, 50 mM DTT, 5 mM EDTA, 0.5% SDS, 0.5% DOC, 5% glycerol, 5 mM benzamidine HCl, 2 mM PMSF and protease inhibitor cocktail (Sigma #P8340). These extracts were used in GST pull-down assays.

### 2.7. Glutathione S-Transferase Pulldown Assay

Equal amounts of recombinant GST and GST–desmin, GST–NDUFS2 or GST–prosaposin were bound to glutathione Sepharose and mixed with either 0.1 mg of desmin-enriched cardiac extract or 1 mg of total protein cardiac extracts or total extracts from transfected COS-7 cells, at room temperature for 2 h. Bound proteins, after washing in the cold washing buffer (10 mM Tris, pH 8.5, 0.01% (*v*/*v*) Nonidet P-40, 0.01% (*v*/*v*) DOC, 5 mM EDTA and 2 mM DTT) were eluted by heating for 5 min at 90 °C in SDS-PAGE sample buffer (50 mM Tris, pH 6.8, 50 mM DTT, 2% (*v*/*v*) SDS, 0.2% (*v*/*v*) bromophenol blue, 10% (*v*/*v*) glycerol). The soluble fractions were analyzed by SDS-PAGE, transferred to a polyvinylidene difluoride membrane, and probed with desmin polyclonal antibody Y-20, (Santa Cruz Biotechnology, Heidelberg, Germany) or anti-NDUFS2 (#PA5-19342, from Thermo Fisher Scientific, Waltham, MA, USA) at a 1:300 dilution. Secondary horseradish peroxidase-conjugated antibodies were from BioRad (Hercules, CA, USA) and visualization of the peroxidase was performed with enhanced chemiluminescence reagents (Amersham Biosciences, Piscataway, NJ, USA).

### 2.8. Immunofluorescent Staining

Frozen rat cardiac tissue sections (12 μm thick) were fixed with acetone/methanol at −20 °C for 20 min, and then used for immunofluorescence staining. Incubations with primary antibodies, namely anti-desmin (DE-U-10 #D1033) from Sigma-Aldrich, anti-NDUFS2 (#PA5-19342) from Thermo Fisher Scientific and anti-saposin D (E14) from Santa Cruz Biotechnology, were performed overnight at 4 °C, at 1:50 dilutions, in 5% BSA in PBS/Tween 20 (0.2%). The appropriate secondary antibodies (conjugated with AlexaFluor-594 and AlexaFluor-488) were obtained from Molecular Probes (Leiden, The Netherlands) and used in 1:1000 dilution. For confocal imaging, a Leica TCS SP5, DMI6000, microscope (inverted, with the acquisition software LAS X Platform, at 23–24 °C; Leica Microsystems, Wetzlar, Germany) was used.

### 2.9. Homology Modeling and Model Evaluation

The homology modeling of the desmin and its interacting molecules was carried out using the MOE (2004.03) package and its built-in homology modeling application. The produced models were initially evaluated within the MOE package by a residue packing quality function, which depends on the number of buried non-polar side chain groups and on hydrogen bonding.

### 2.10. Molecular Electrostatic Potential (MEP)

Electrostatic potential surfaces were calculated by solving the nonlinear Poisson–Boltzmann equation using the finite difference method as implemented in the PyMOL-Software (DeLano, W.L. The PyMOL Molecular Graphics System, Schrodinger, LLC, New York, NY, USA; http://www.pymol.org). The potential was calculated on grid points per side (65, 65, 65) and the ‘grid fill by solute’ parameter was set to 80%. The dielectric constants of the solvent and the solute were set to 80.0 and 2.0, respectively. An ionic exclusion radius of 2.0 Å, a solvent radius of 1.4 Å and a solvent ionic strength of 0.145 M were applied. AMBER99 charges and atomic radii were used for this calculation.

### 2.11. Docking Studies and Protein–Protein Interactions

The docking studies amongst the various constructed models or crystal structures were executed using ZDOCK version 3.0 [30]. Likewise, RDOCK was used in order to minimize the ZDOCK complex outputs and re-rank them based on their re-estimated binding free energies. Upon docking experiments, all molecular systems were subjected to extensive energy minimizations up to a Gradient G < 0.0001, using the Charmm27 forcefield as implemented into the Gromacs 4.5.5 suite, using our in-house developed graphical interface.

### 2.12. Energy Minimizations before Dynamics

Energy minimization was performed initially to remove any remaining geometrical strains in the structure of the PDB coordinate file. Protein complexes were subjected to an extensive energy minimization run using the Amber99 [31] force field, as it is implemented into the Gromacs via our previously developed graphical interface for it [32]. An implicit Generalized Born (GB) solvation was chosen at this stage, in an attempt to speed up the energy minimization process.

### 2.13. Molecular Dynamics

In order to further explore the interaction space and binding potential, the docked molecular complexes were subjected to unrestrained molecular dynamics simulations using the Gromacs suite, version 4.5.5. Molecular dynamics took place in a periodic environment, which was subsequently solvated with SPC water using the truncated octahedron box extending to 7 Å from each molecule. Partial charges were applied, and the molecular systems were neutralized with counter-ions as required. The temperature was set to 300 K, pressure at 1 atm and the step size was set to 2 femtoseconds. Herein, a script was used to heat the system from 0 to 300 K, followed by an equilibration and a production phase. The last step involved the cooling down of the system from 300 K to 0 K. The heating stage was set to 10 nanoseconds, which was then followed by an equilibration phase run for each molecular complex for another ten nanoseconds, using the NVT ensemble in a canonical environment. The equilibration phase of each molecular system was then followed by a five hundred nanosecond production phase using the NVT ensemble. NVT stands for Number of atoms, Volume and Temperature, which remain constant throughout the calculation. Finally, each molecular system was cooled down from 300 K to 0 in a cooling stage for another ten nanoseconds. The results of the molecular dynamics simulations were collected into a molecular trajectory database and can be further analyzed.

### 2.14. Post Molecular Dynamics Analysis

Principal component analysis was carried out using Pymol (DeLano, W.L. The PyMOL Molecular Graphics System, Schrodinger, LLC; http://www.pymol.org) and the Ca atom root mean square function of Deep-View [33]. Analysis of the molecular dynamics outputs and trajectories was therefore focused on the structural deviations of each molecular system from its original conformation. The molecular dynamics final conformations were initially evaluated with a residue packing quality function built into the Gromacs suite, which depends on the number of buried non-polar side chain groups and on hydrogen bonding. Moreover, the suites Procheck [34] and Verify3D [35] were employed to evaluate the structural viability of each protein complex upon the molecular dynamics simulations. Illustrations of the molecular systems were rendered with the aid of the Chimera suite [36].

## 3. Results

### 3.1. Desmin Binds to Mitochondrial and Lysosomal Proteins NDUFS2 and Saposin D, Respectively

**Figure 1:**
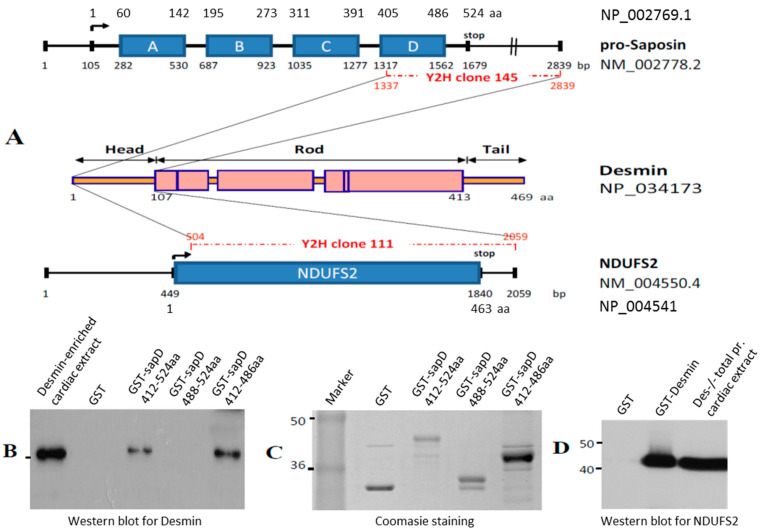
NDUFS2 and Saposin D identified as desmin’s head domain binding partners. (**A**) Schematic representation of clone-111 (NDUFS2, GenBank accession number NM_004550; fragment 504–2059 bp) and clone-145 (pro-saposin D, GenBank accession number NM_002778; fragment 1337–2839 bp), identified using yeast two-hybrid system as desmin head domain binding proteins. (**B**) Specific binding of desmin to GST–saposin D. Desmin-enriched cardiac extracts (as a source of desmin, lane 1) and equivalent amounts of GST (lane 2), GST–saposin D (lane 3) and GST–saposin D fragments (lanes 4 and 5) expressed in bacteria were bound to glutathione Sepharose. Recombinant GST–saposin D fragment 412–524 aa and the GST–saposin D fragment 412–486 aa, but not GST or GST–saposin D fragment 488–524 aa, absorbed desmin, as indicated by Western blot analysis (aa numbering is from NCBI Reference Sequence: NP_002769.1). (**C**) Coomassie staining of the bound to glutathione Sepharose proteins from panel B, eluted by SDS. (**D**) Specific binding of NDUFS2 to GST–desmin. Equivalent amounts of GST (lane 1) and GST–desmin (lane 2) expressed in bacteria were bound to glutathione Sepharose. Recombinant GST–desmin, but not GST alone, absorbed NDUFS2 from total protein cardiac extracts preparation, as indicated by Western blot analysis. Total protein cardiac extracts from desmin null (Des−/−) mice (lane 3) used as a source of NDUFS2 were also analyzed.

In an effort to explore the role of the desmin head domain, a human cardiac cDNA library was screened using the yeast two-hybrid system, in order to identify potential binding partners. The amino terminal domain (1–103 aa) of the desmin molecule was used as a “bait” to screen approximately 1 × 10^6^ transformants, of which 170 “prey” clones met the stringent criteria as potential desmin binding partners, as we have previously described [28]. After retesting each one of the isolated positive clones by co-transformation into AH109 cells on SD/–Ade/–His/–Leu/–Trp/X-α-Gal plates and analyzing them using yeast colony PCR to eliminate duplicated clones, 22 potential desmin head binding partners were identified (Supplementary Table S1). From the sequencing analysis, clone-111 was identified as NADH ubiquinone oxidoreductase core subunit S2 (NDUFS2, GenBank accession number NM_004550; fragment 504–2059 bp) and clone-145 as pro-saposin D (GenBank accession number NM_002778; fragment 1337–2839 bp) (Figure 1A). NDUFS2 was a core subunit of the mitochondrial membrane respiratory chain NADH dehydrogenase (complex I) and the pro-saposin gene encoded a highly conserved glycoprotein which was a precursor for four cleavage products, saposins A, B, C and D, which localized primarily to the lysosomal compartment. Clone-145 covered most of the saposin D sequence (Figure 1A).

Confirmation of desmin–NDUFS2 or –saposin D interaction was achieved by GST pull-down assays. Recombinant GST–saposin D or GST–desmin were prepared in bacteria and used for binding studies to desmin (Figure 1B) or NDUFS2 (Figure 1D), respectively, using cardiac extracts as a source for these proteins. Desmin or NDUFS2 were efficiently absorbed to GST–saposin D or GST–desmin, respectively, bound to a glutathione matrix, as detected by Western blot analysis using anti-desmin or anti-NDUFS2 antibodies (Figure 1B,D). The inability of GST alone to absorb desmin or NDUFS2 confirmed the specificity of this interaction (Figure 1B,D). The desmin–saposin D interaction was also demonstrated by coimmunoprecipitation analysis of the in vitro transcribed/translated products (Supplementary Figure S1). To further determine the precise corresponding region of saposin D necessary and sufficient for interaction with desmin, the GST pull-down assays were extended by generating saposin D deletion constructs. Two constructs were generated, saposin D-(1569–2839 bp), which encodes for the last 36 amino acids (488–524 aa) of the prosaposin molecule, necessary for the sorting and targeting of prosaposin to lysosomes [37], and saposin D (1337–1564 bp) fragment which encodes for the 74 aa (412–486 aa) of the 81 saposin D amino acids, responsible for its activity. Specific binding was observed only for the saposin-D 1337–1564 bp (412–486 aa) fragment (Figure 1B). To further analyze the desmin head interactions with NDUFS2 or saposin D at an atomic level, an in silico approach, using a desmin 3D structure and molecular docking analysis, was performed.

### 3.2. In Silico Analysis of Interactions

#### 3.2.1. Three-Dimensional Model of Head Domain of Desmin

The bacterial crystal structure of methylmalonyl-Coa mutase was used as a template for the homology modeling of desmin’s head domain (RCSB entry: 1 REQ). The sequence identity and similarity were 31% and 44%, respectively, which marginally allowed for conventional homology protocols to be considered. The homology modeling algorithm of the MOE (2004.03) package was used, as described in the Section 2. The algorithm worked in a three-step pipeline, as follows. First, an initial partial geometry specification step was undertaken, where an initial partial geometry for each target sequence was copied from regions of one or more template chains. Second, the insertions and deletions task was undertaken, where residues that still had no assigned backbone coordinates were modelled. Third, the loop selection and sidechain packing step was undertaken, where a collection of independent models was created. The last step was the final model selection and refinement step, where the final models were scored and ranked, after they had been stereochemically tested and evaluated with the built-in module “Protein Geometry” for errors. The model was firstly structurally superimposed and subsequently compared to its template, where it exhibited an alpha-carbon RMSD less than 0.55 angstroms. 

**Figure 2:**
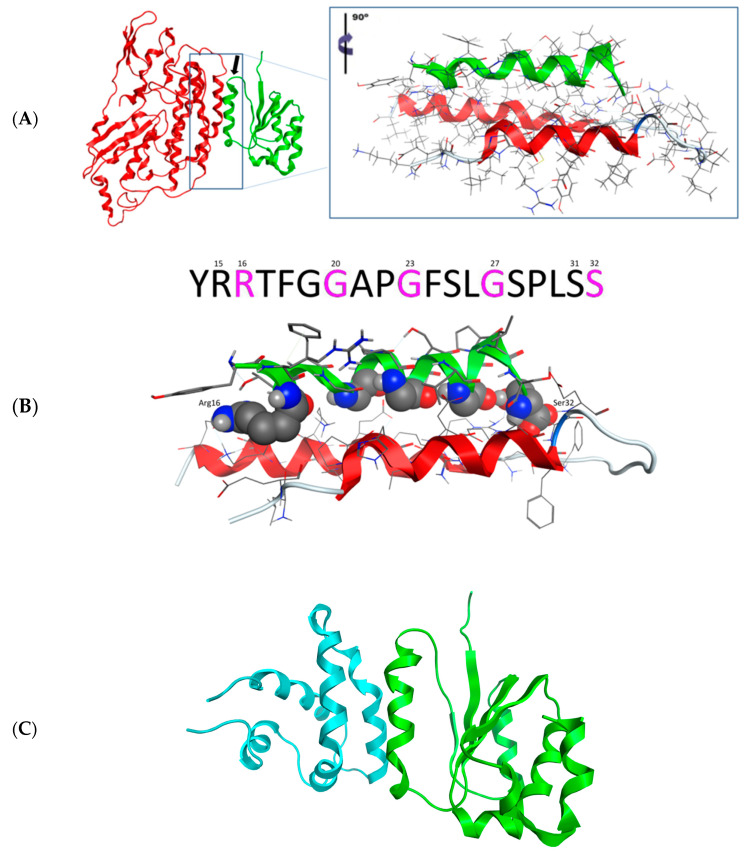
(**A**) The docking pose between the homology models of desmin’s head domain (represented by the green ribbon) and NDUFS2 protein (represented by the red ribbon). The insert focuses on the three-helix bundle that is formed by the interaction of the two proteins. The interacting α-helix from desmin’s head is shown with a black arrow. The insert has been tilted 90° from the vertical axis. (**B**) The interaction between desmin’s head and NDUFS2. The interacting residues from desmin’s head domain are showing in space fill representation. Notably, Arg16 lies next to Arg15 and Ser32 is next to Ser31. This is a safety measure that ensures the viability of the hydrogen bonding interaction at the event of mutation or loss of either Arg16 or Ser32 residues. The core of desmin’s head domain interacting α-helix is lined by a series of three glycines (Gly20, Gly23 and Gly27), which contribute both sterically and electrochemically to the stabilization of the interaction. (**C**) The interaction between desmin’s head domain and saposin D protein. Desmin’s head 3D homology model used in this docking experiment is represented by the green ribbon, whereas the X-ray crystal structure of saposin D (RCSB entry: 3 BQP) is represented by the blue ribbon.

The 3D homology model of desmin’s head domain consisted of a series of alpha helical motifs that surrounded a central motif consisting of two parallel beta sheets. The majority of the alpha helices were rather short in length, ranging from a mere 5–6 to even 10–12 amino acids. There was only one exception: a single α-helix that was considerably longer than the rest on the modeled 3D structure of desmin’s head domain (Figure 2A, represented by the green ribbon).

#### 3.2.2. Three-Dimensional Model of NDUFS2 and the Saposin-D X-ray Crystal Structure

The homology model of clone-111 (NDUFS2, NM_004550) was conducted in a similar way to that previously described for the desmin head domain. The crystal structure of the hydrophilic domain of respiratory complex I from Thermus Thermophilus (RCSB entry: 2 FUG) was used as a template. The sequence identity and similarity were 46% and 66%, respectively. The modeled 3D structure of NDUFS2 is shown in Figure 2A,B (represented by the red ribbon). On the other hand, it was found that the 3D structure of the clone-145 (prosaposin, NM_002778) protein was determined by X-ray crystallography (RCSB entry: 3 BQP). Only part of the full prosaposin protein has been crystallized and structurally determined by X-ray crystallography. The corresponding nucleotide sequence ranges from 1337 to 1562 bases, which covers the saposin D protein that was found to interact with the desmin head domain by the yeast to hybrid and GST pull-down assays. The produced 3D model of NDUFS2 (Figure 2A, represented by the red ribbon) and the X-ray crystal structure of saposin D (Figure 2C, represented by the blue ribbon) were both prepared for the docking experiments with desmin’s head domain 3D model.

#### 3.2.3. Molecular Docking of the Three-Dimensional Desmin Head Domain and NDUFS2

Molecular docking experiments were conducted on a two-step basis. First, the Zdock rigid body docking algorithm was employed to screen for plausible acceptable conformation between the desmin’s head and its molecular partners. Then, an unconstrained energy minimization algorithm was applied to each molecular complex system, in an effort to eradicate any residual geometrical strain from the previous docking phase.

**Figure 3:**
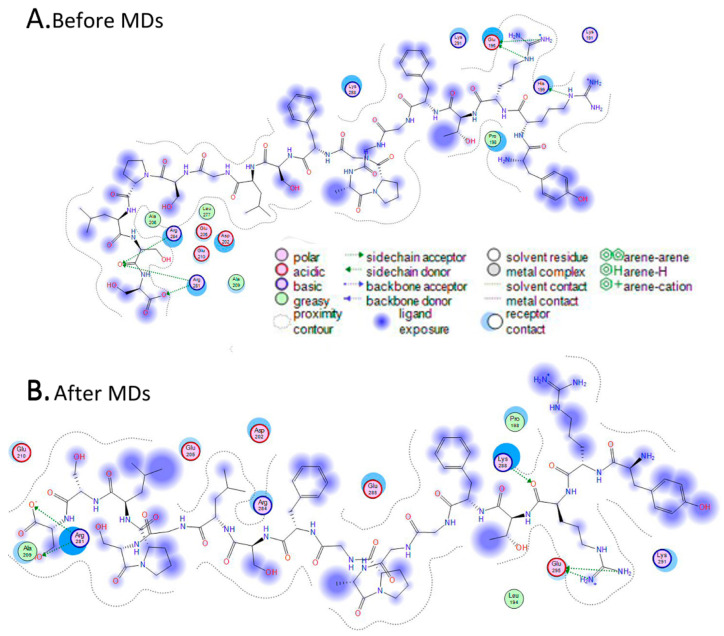
Two-dimensional Ligplot representation of the interaction established between desmin’s head domain and NDUFS2 protein. (**A**) This is the specific interactions that were established after the docking experiment, before the molecular dynamics simulation (MDs). As depicted, the only hydrogen bonding interactions are located on the outer sides of desmin’s interacting α-helix. (**B**) Same as (**A**), only this is the interaction after the molecular dynamics simulation. It is clear that the hydrogen bonds successfully retained their strength/position upon the molecular dynamics simulation. Note that this interaction map has been horizontally flipped by Ligplot.

The docking experiment returned only one viable solution as a plausible interaction between desmin’s head domain and NDUFS2 (Figure 2A,B). This single pose involved the establishment of a three-helix bundle, comprising one α-helix from desmin and two α-helical domains from the NDUFS2 model. The interaction was made up from a series of both hydrogen bonding and hydrophobic interactions. The molecular system that was already energetically minimized was subjected to the MOE’s built-in Ligplot 2D molecular visualization module. It was found that the only stabilizing hydrogen bonds were established on either end of the desmin’s head α-helix, with the NDUFS2 residues in close proximity (Figure 3B). The main body of the interaction was stabilized by hydrophobic interactions formed in the interdomain space of the three-helix bundle.

In an effort to solidify this argument, the energy-minimized molecular complex was put to the test, by subjecting it to molecular dynamics (MDs) simulations. Examination of the MDs molecular output confirmed the pattern of interaction as previously described. Desmin established a hydrogen bond between Arg16 and His199 from NDUFS2 (renumber from the 2FUG_4, NM_004550). Likewise, another hydrogen bond was established between Ser32 and Arg281 and Arg284 (renumbered from the 2FUG_4, NM_004550). Notably, desmin has two identical polar residues on either end of the interacting α-helix. This is believed to be a conservation mechanism against random malfunctions or mutations that would disrupt the interacting properties and capabilities of the desmin’s head domain. The rest of the interacting residues of desmin’s α-helix are a well-organized structure of small glycine amino acids. Their role is very crucial to the establishment of the molecular interaction, as they have small side chains that enable the indispensable close packing of the helices together and promote the hydrophobic associations that stabilize the molecular system.

#### 3.2.4. Molecular Docking of the Three-Dimensional Desmin Head Domain and Saposin D

**Figure 4:**
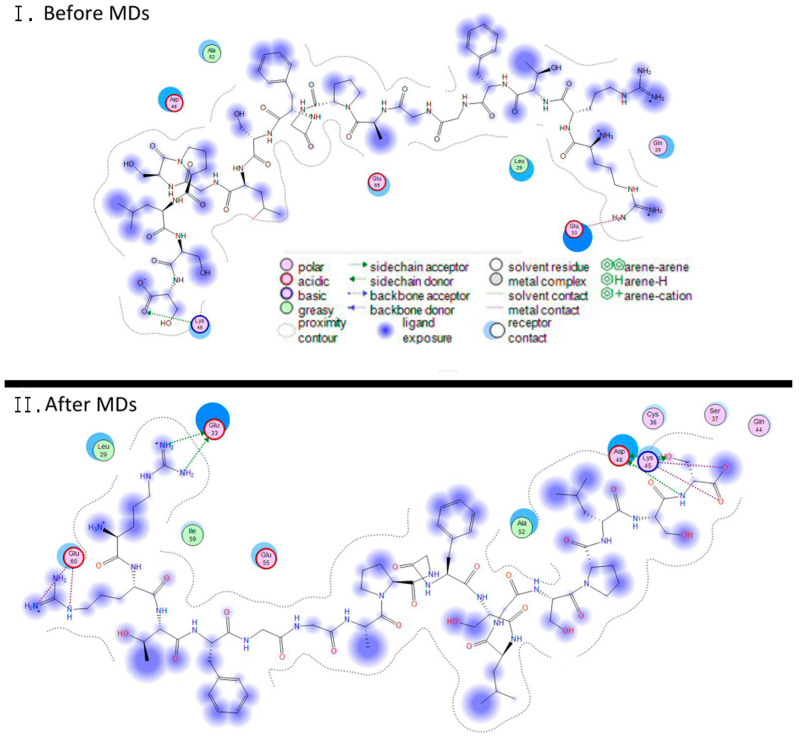
A 2D Ligplot representation of the interaction established between the desmin’s head and saposin-D. (**I**) These are the specific interactions that were established after the docking experiment, before the molecular dynamics (MDs) simulation. The only hydrogen bonding interactions are between Arg16 and Glu33 and Ser32 and Lys45/Asp48 (renumbered from the 3BQP_B, NM_002778). (**II**) Same as (**I**), except these are the interactions after the molecular dynamics simulation.

The molecular docking experiment of the desmin head domain to saposin D returned a single pose as a viable conformation that favors the interaction of the two structures. The 3D structure of prosaposin-D has been determined via X-ray protein crystallography, which dramatically increases the reliability of the docking experiment. The single pose of interaction follows the same pattern of the desmin head domain and saposin D complex with the formation of a three-helix bundle that is stabilized by a combination of hydrogen bonding and hydrophobic interactions (Figure 2C and Figure 4). The hydrogen bonds are located in a strategic formation that outflanks the interacting α-helix of desmin’s head domain. The core of this protein–protein interaction is mainly hydrophobic interactions. 

#### 3.2.5. Both Interactions Occur in a Very Similar Pattern

**Figure 5:**
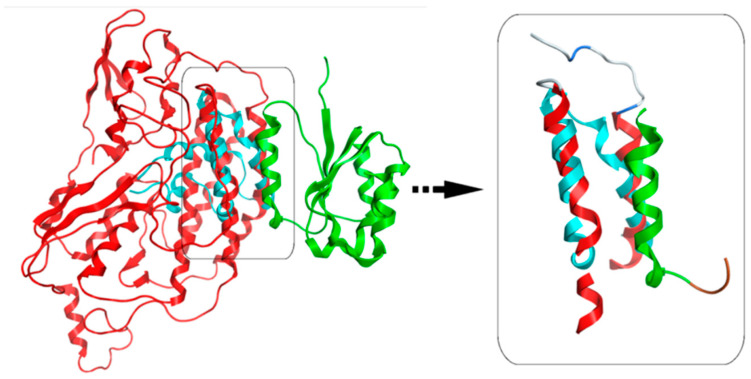
Structural superposition of the two docked molecular complexes. The common structures of NDUFS2 (red ribbon) and saposin D (blue ribbon) interacting with desmin’s head domain (green ribbon) were superposed (right panel). It was found that the NDUFS2 3D homology model and the saposin D X-ray structure share identical 3D conformations that favor the specific interaction with the active modeled conformation of desmin’s head domain. Note that this interaction map has been horizontally flipped by Ligplot.

Since the interaction patterns of NDUFS2 and saposin D with desmin’s head domain were of a very similar nature, we superimposed the desmin molecules from each individual docking experiment and studied the relative positioning of the docked NDUFS2 and saposin D molecules. Strikingly, it was found that the interacting parts of both molecules (NDUFS2 and saposin D) with desmin were almost identical, as, for this part, they share a Ca root mean squared deviation (RMSd) less than 1 Å. This is a very important finding as it both rationalizes the mode of 3D spatial conformation that needs to be adopted for successful interaction and the fact that NDUFS2 and saposin D share identical regions interacting with desmin (Figure 5). The latter adds up to the confirmation of the reliability of our 3D homology model of the NDUFS2 protein, since its identical saposin D counterpart is an actual crystal structure of high reliability (solved at a resolution less than 1.30 Å). 

#### 3.2.6. Prediction of Desmin’s Head Domain Three-Dimensional Structure by AlphaFold

**Figure 6:**
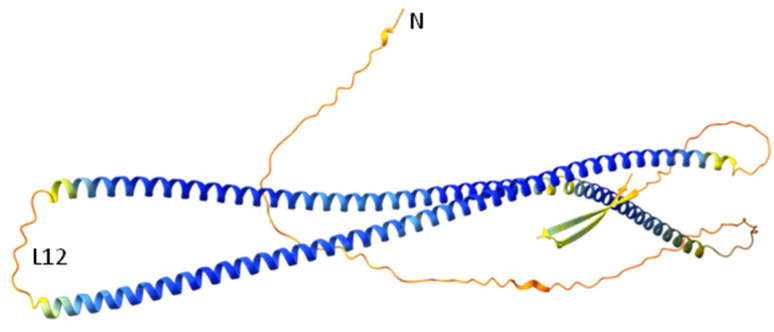
Prediction of desmin’s 3D structure by AlphaFold. The α-helical rod domain intervenes by the linker L12. Head and tail domains seem mainly unstructured, with the exception of a β-strand formation on the tail. Model confidence/color: very high (pLDDT > 90)/blue; high (90 > pLDDT > 70)/light blue; low (70 > pLDDT > 50)/yellow and very low (pLDDT < 50)/orange. (N: amino terminus).

The prediction of desmin’s 3D structure by AlphaFold [38,39] showed a lack of specific structure on the head domain (Figure 6), although the per-residue confidence score (predicted local distance difference test, pLDDT) was very low (<50), so it is not considerable. It is suggested that regions with pLDDT < 50 were maybe either un-structured in physiological conditions or only structured as part of a complex [38,39], and maybe that was the case in the process of IF assembly.

### 3.3. Desmin Head Deletion Abolishes Interaction with NDUFS2 and Saposin-D

**Figure 7:**
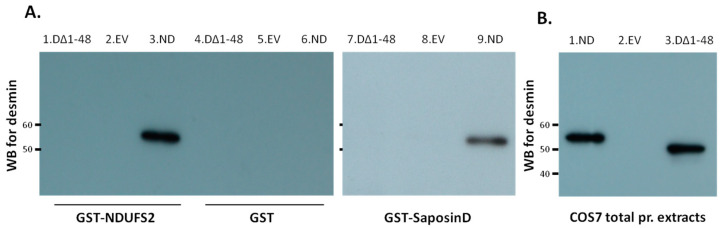
(**A**) No binding of headless desmin to GST–NDUFS2 and GST–saposin D. Equivalent amounts of GST–NDUFS2 (lanes 1, 2 and 3), GST (lanes 4, 5 and 6) and GST–saposin D (lanes 7, 8 and 9) expressed in bacteria were bound to glutathione Sepharose. Only the normal desmin (ΝD, lanes 3 and 9), but not the headless desmin (DesminΔ1-48) (lanes 1 and 7), was absorbed by recombinant GST–NDUFS2 and GST–saposin D, as indicated by Western blot (WB) analysis. (**B**) Western blot analysis of desmin of total protein extracts of COS7 cells transfected with normal desmin, empty vector or desminΔ1-48 (lanes 1, 2 and 3, respectively) used for the GST pull-down assays. Abbreviations: DΔ1-48: desmin with 1-48 aa deletion, EV: empty vector and ND: normal desmin.

Following the previous results, we wanted to confirm in vitro that the interactions of desmin with NDUFS2 and saposin D were essentially mediated by the head domain. Recombinant GST–NDUFS2 or GST–saposin D expressed in bacteria were prepared and used for binding studies on protein extracts from COS7 cells transfected with head-less desmin (desminΔ1-48) or either an empty vector (EV, negative control) or normal desmin (ND, positive control) (Figure 7). Only the wild-type desmin was efficiently absorbed by GST–NDUFS2 or GST–saposin D bound to a glutathione matrix, and not the head-less desmin (desminΔ1-48), as shown by Western blot analysis using an anti-desmin antibody (Figure 7A). The inability of GST alone to absorb desmin of any kind confirmed the specificity of this interaction (Figure 7A).

### 3.4. Desmin Partially Co-Localizes with NDUFS2 and Saposin D in Cardiomyocytes

**Figure 8:**
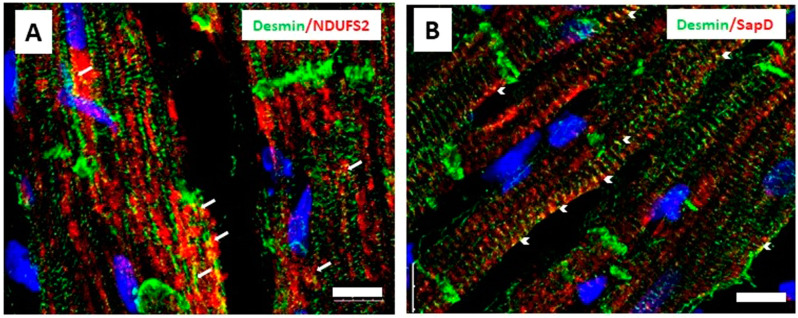
Immunofluorescence staining of cardiac tissue sections for desmin (green) and NDUFS2 (red) (**A**), indicating partial co-localization in areas close to sarcolemma and nucleus (arrows) and (**B**) for desmin and saposin D (red) at z-lines close to sarcolemma, presumably at costameres (arrowheads). (Blue: DAPI staining of nuclei, images are projections of confocal images; scale bar 10 μm)**.**

In order to integrate the in silico and the in vitro results indicating the interactions of desmin with NDUFS2 and saposin D, we stained cardiac tissue sections for desmin and NDUFS2 or saposin D. The partial co-localization of desmin and NDUFS2 was observed at areas close to the sarcolemma and nucleus (Figure 8A) and for desmin and saposin D at costameres, at the level of z-lines (Figure 8B).

## 4. Discussion

The present work, to the best of our knowledge, is the first report demonstrating, using in silico analysis at an atomic level and in vitro using GST pull-down assays, the interaction of NDUFS2 and saposin D with desmin. The mitochondrial protein NDUFS2 is one of the core subunits of the largest multimeric enzyme complex of the mitochondrial respiratory chain, complex I, and specifically is a subunit of the Q module, which is responsible for the electron transfer to ubiquinone. Mitochondrial complex I is responsible for the oxidation of NADH. NDUFS2 is very important for complex I assembly, as, in the early assembly stage, it forms a small hydrophilic assembly complex with NDUFS3 [40]. Recently, desmin was found to interact with Mic60, the core component of MICOS (the mitochondrial contact site and cristae organizing system) [41]. They suggested that the demonstrated associations of desmin with VDAC, Mic60 and ATP synthase could somehow facilitate the formation and/or stabilization of a scaffold-like super complex, extending from the endoplasmic reticulum–mitochondria contact sites to MICOS and ATP complexes [41]. In the same way, a desmin cytoskeleton may act as a scaffold for the proper formation of complex I and sort its nucleus-encoded proteins, like NDUFS2, into mitochondria. A recent study showed that desmin, especially the pure recombinant DesY122L, binds directly to isolated rat liver mitochondria and that the presence of the head domain is necessary for this interaction [42]. In agreement with this, immunofluorescence staining of rat cardiac tissue sections showed partial co-localization for desmin and NDUFS2 in areas close to the sarcolemma and nucleus. The ablation of desmin in mouse results in characteristic alterations in the distribution, number, morphology and respiratory activity of mitochondria [43,44,45,46]. Complex I deficiency is the most common respiratory chain defect. Mutations in NDUFS2 are related to mitochondrial complex I deficiency and Leigh syndrome [47] or cardiomyopathy and encephalomyopathy [48]. Recent studies indicate that NDUFS2 is essential for acute oxygen-sensing and hypoxic pulmonary vasoconstriction, affecting ROS and calcium signaling [49] or could be related to cancer, since the inhibition of complex I through NDUFS2 leads to anti-cancer activity by targeting cancer energy metabolism [50,51].

Another mitochondrial binding partner of desmin’s head domain, identified by the yeast two-hybrid assay, is cytochrome c oxidase subunit I (COXI) (clone-143, Supplementary Table S1), which is the primary subunit of the catalytic core of the mitochondrial respiratory chain enzyme, complex IV. The mitochondrial complex IV is also a multimeric enzyme, but of dual genetic origin, as the subunits COXI, COXII and COXIII are encoded by mitochondrial DNA and the remaining subunits are encoded by nuclear DNA. COXI and COXII are fundamental to the proper assembly of complex IV [52]. COX deficiency is characterized by a high degree of genetic and phenotypic heterogeneity and is correlated with metabolic abnormalities and several diseases like Leigh syndrome and myopathies [53]. In addition, the yeast two-hybrid assay indicated that desmin’s head domain also interacts with aldehyde dehydrogenase 4 family member A1 (ALDH4A1) (clone-132, Supplementary Table S1), which is a mitochondrial enzyme involved in proline metabolism [54] and is related to atherosclerosis [55]. A potential role of intermediate filaments in affecting mitochondrial structure and function is becoming more apparent [56].

Additionally, we showed that the desmin head domain interacts with the lysosomal protein prosaposin and, specifically, saposin D. Saposins act as activator proteins and promote the function of hydrolases involved in sphingolipid degradation. Saposin D is an acid ceramidase activator, and mice with saposin D deficiency show disturbed ceramide metabolism and cellular degenerations [57]. Prosaposin deficiency leads to a complex neurodegenerative phenotype with sphingolipid accumulation [58]. Recent studies relate saposin D to Parkinson disease, due to lysosome storage disorders [59]. Furthermore, secretory prosaposin affects the progression of prostate [60], breast [61] and glioma [62] cancer. Regarding the transportation of prosaposin to lysosomes, the interactions of a highly conserved saposin D domain with sphingomyelin [63] and the carboxy terminal of prosaposin (PMBD domain) to sortilin [37] are necessary. Our findings may suggest that the desmin cytoskeleton is involved in the sorting and trafficking of prosaposin to lysosomes in a similar way to the mitochondrial proteins. Studies of desmin-deficient hearts suggest that desmin has a role in lysosome and lysosome-related organelle biogenesis and positioning [28]. In fact, desmin binds to myospryn [28], which, in turn, can interact with dysbindin, a component of the biogenesis of lysosome-related organelles complex 1 (BLOC-1), which regulates protein trafficking and organelle biogenesis [64]. Myospryn and dysbindin also interact with the dystrophin-associated glycoprotein complex (DGC) [65,66], which is localized at the sarcolemma at the level of costameres. Correspondingly, immunofluorescence staining of rat cardiac tissue sections showed co-localization for desmin and saposin D at z-lines close to the sarcolemma, presumably at costameres. Myospryn deficiency is associated with cardiac and brain disorders [67]. Another lysosomal binding partner of desmin’s head domain that we observed using the yeast two-hybrid system is cathepsin D (clone-158, Supplementary Table S1). Cathepsin D is a lysosomal protease important for the degradation of various substrates, as is the neurodegenerative diseases-associated protein α-synuclein [68]. Cathepsin D participates in prosaposin cleavage too [69], and, together with progranulin, participates in a lysosomal network involved in Parkinson’s [70].

In conclusion, the head domain of desmin, through its interactions, may play a role in the proper subcellular positioning and maintenance of mitochondria and lysosomes, such as in the proper assembly and integrity of complexes. Alterations in protein interactions may affect specific subcellular functions, leading to disease development. 

### Bioinformatic Analysis

Subsequently, we proceed to protein–protein interaction analysis, not only for analyzing whether these proteins interact, but also to gain an insight into the physicochemical profile and residues (sites) of the protein interface. Interestingly, the interactions occur in a very similar way, indicating a pattern. Specifically, the interaction of the 3D model of the head domain of desmin with the NDUFS2 3D model requires the establishment of a three-helix bundle, which comprises one α-helix from desmin and two α-helical domains from the NDUFS2 model and is stabilized by a combination of hydrogen bonding and hydrophobic interactions, as well as with the saposin D X-ray crystal structure, which improves the reliability of the docking experiment. Certainly, the residues that contribute to both interactions on the α-helix of desmin side are Arg16 and Ser32. Both residues are related to cardiomyopathies in humans [19,27], and Ser32 is also a phosphorylation site, which contributes to desmin assembly and cardiac toxic PAOs deposition [21].

Last but not least, the 3D model of the desmin head domain revealed that it consists of a series of alpha helical motifs that surround a central motif of two parallel beta sheets, which contradicts the previous theory that assumed that the head domain was unstructured and of a low level of complexity. Until a reliable possible crystallographic study reveals its structure in nature, the theory will be controversial. Not surprisingly, the crystallization of IF fragments containing poorly ordered parts has proven to be a challenge. However, in tail domain of lamin A/C, an Ig-like domain with a β-sheet formation in dimer has been characterized [7,8]. Also, two short overlapping fragments of the vimentin head domain (residues 59–71 and 66–78) have been crystallized recently, when bound to a human leukocyte antigen (PDB codes 4MD0 and 4MDJ) [71]. At the same time, some information on the terminal domains has been gathered, using SDSL-EPR as well as chemical cross-linking [2], allowing the creation of initial models indicating their overall topology [12,72]. In particular, within an individual vimentin dimer, the head domains appear to fold back onto one side of the coil1 domain, bringing residues 17 and 137 into close proximity, breaking the overall symmetry of the dimer [12]. This asymmetry may help to differentiate between possible inter-dimeric interactions and, thus, contribute to defining a specific pathway of filament assembly [73]. Furthermore, it has been noted that residues 12, 17, 32 and 83 move farther apart upon phosphorylation [12,13] and maybe affect our interactions. A recent study reinforces the importance of the interaction between the head and rod domains for the proper formation of vimentin intermediate filaments, as, when this interaction was hindered by N-terminal tags, the N-terminally tagged vimentin form aggregated, while the mixed tagged and untagged vimentin formed a less stable network [74].

Interestingly, the prediction of the desmin 3D structure by AlphaFold shows a lack of specific structure on the head’s domain, although the per-residue confidence score (predicted local distance difference test, pLDDT) is very low (<50), so it is not considerable [38,39]. It should be also mentioned that IFs seems to exhibit structural diversity [75,76,77]. Our 3D model of desmin’s head domain shows a structural order consisting of both alpha helical motifs and beta sheets. In comparison, the prediction of AlphaFold for desmin shows beta sheet formation in the tail domain, which maybe became from the crystal structure of the homologous lamin’s tail domain [8]. Therefore, more studies are needed to understand the actual structure of the IF proteins’ head domains and the process of IF assembly.

## 5. Conclusions

Desmin interacted in vitro, with the mitochondrial protein NDUFS2 and the lysosomal protein saposin D, as analyzed by using GST pull- down analysis. More specifically, desmin’s head domain was necessary for both interactions. The in silico analysis revealed that the pattern of interaction was similar in both molecular interactions. In addition, the yeast two-hybrid assay indicated that desmin’s head domain could interact with additional mitochondrial and lysosomal proteins. Through those interactions, desmin may be involved in mitochondria and lysosome cellular functions, which could lead to different type of diseases when disrupted.

## Data Availability

The original contributions presented in the study are included in the article/Supplementary Materials, further inquiries can be directed to the corresponding author.

## Supplementary Materials

The following supporting information can be downloaded at the following link: https://www.mdpi.com/article/10.3390/cells13070603/s1, Table S1. Desmin head domain binding partners identified by the yeast two-hybrid system. Figure S1: direct interaction of desmin with saposin D. Coimmunoprecipitation analysis of desmin and saposin D labelled with [^35^S] methionine with a TNT Quick Coupled Transcription/Translation System.

## References

[1] Chernyatina A.A., Nicolet S., Aebi U., Herrmann H., Strelkov S.V. (2012). Atomic structure of the vimentin central α-helical domain and its implications for intermediate filament assembly. Proc. Natl. Acad. Sci. USA.

[2] Herrmann H., Aebi U. (2004). Intermediate filaments: Molecular structure, assembly mechanism, and integration into functionally distinct intracellular Scaffolds. Annu. Rev. Biochem..

[3] Nicolet S., Herrmann H., Aebi U., Strelkov S.V. (2010). Atomic structure of vimentin coil 2. J. Struct. Biol..

[4] Herrmann H., Strelkov S.V. (2011). History and phylogeny of intermediate filaments: Now in insects. BMC Biol..

[5] Herrmann H., Häner M., Brettel M., Müller S.A., Goldie K.N., Fedtke B., Lustig A., Franke W.W., Aebi U. (1996). Structure and Assembly Properties of the Intermediate Filament Protein Vimentin: The Role of its Head, Rod and Tail Domains. J. Mol. Biol..

[6] Schaffeld M., Herrmann H., Schultess J., Markl J. (2001). Vimentin and desmin of a cartilaginous fish, the shark Scyliorhinus stellaris: Sequence, expression patterns and in vitro assembly. Eur. J. Cell Biol..

[7] Gruenbaum Y., Foisner R. (2015). Lamins: Nuclear intermediate filament proteins with fundamental functions in nuclear mechanics and genome regulation. Annu. Rev. Biochem..

[8] Ahn J., Lee J., Jeong S., Kang S.-M., Park B.-J., Ha N.-C. (2021). Beta-strand-mediated dimeric formation of the Ig-like domains of human lamin A/C and B1. Biochem. Biophys. Res. Commun..

[9] Zhou X., Lin Y., Kato M., Mori E., Liszczak G., Sutherland L., Sysoev V.O., Murray D.T., Tycko R., McKnight S.L. (2021). Transiently structured head domains control intermediate filament assembly. Proc. Natl. Acad. Sci. USA.

[10] Eibauer M., Weber M.S., Kronenberg-Tenga R., Beales C.T., Boujemaa-Paterski R., Turgay Y., Sivagurunathan S., Kraxner J., Köster S., Goldman R.D. (2023). Vimentin filaments integrate low complexity domains in a highly complex helical structure. bioRxiv.

[11] Zhou X., Kato M., McKnight S.L. (2023). How do disordered head domains assist in the assembly of intermediate filaments?. Curr. Opin. Cell Biol..

[12] Aziz A., Hess J.F., Budamagunta M.S., Voss J.C., FitzGerald P.G. (2010). Site-directed Spin Labeling and Electron Paramagnetic Resonance Determination of Vimentin Head Domain Structure*. J. Biol. Chem..

[13] Aziz A., Hess J.F., Budamagunta M.S., FitzGerald P.G., Voss J.C. (2009). Head and Rod 1 Interactions in Vimentin*. J. Biol. Chem..

[14] Guharoy M., Szabo B., Contreras Martos S., Kosol S., Tompa P. (2013). Intrinsic structural disorder in cytoskeletal proteins. Cytoskelet.

[15] Kornreich M., Avinery R., Malka-Gibor E., Laser-Azogui A., Beck R. (2015). Order and disorder in intermediate filament proteins. FEBS Lett..

[16] Beuttenmüller M., Chen M., Janetzko A., Kühn S., Traub P. (1994). Structural elements of the amino-terminal head domain of vimentin essential for intermediate filament formation in vivo and in vitro. Exp. Cell Res..

[17] Kaufmann E., Weber K., Geisler N. (1985). Intermediate filament forming ability of desmin derivatives lacking either the amino-terminal 67 or the carboxy-terminal 27 residues. J. Mol. Biol..

[18] Goldman R.D., Cleland M.M., Murthy P., Mahammad S., Kuczmarski E.R. (2012). Inroads into the Structure and Function of Intermediate Filament Networks. J. Struct. Biol..

[19] Agnetti G., Halperin V.L., Kirk J.A., Chakir K., Guo Y., Lund L., Nicolini F., Gherli T., Guarnieri C., Caldarera C.M. (2014). Desmin modifications associate with amyloid-like oligomers deposition in heart failure. Cardiovasc. Res..

[20] Sugden P.H., Fuller S.J., Weiss S.C., Clerk A. (2008). Glycogen synthase kinase 3 (GSK3) in the heart: A point of integration in hypertrophic signalling and a therapeutic target? A critical analysis. Br. J. Pharmacol..

[21] Rainer P.P., Dong P., Sorge M., Fert-Bober J., Holewinski R.J., Wang Y., Foss C.A., An S.S., Baracca A., Solaini G. (2018). Desmin Phosphorylation Triggers Preamyloid Oligomers Formation and Myocyte Dysfunction in Acquired Heart Failure. Circ. Res..

[22] Geisler N., Weber K. (1988). Phosphorylation of desmin in vitro inhibits formation of intermediate filaments; identification of three kinase A sites in the aminoterminal head domain. EMBO J..

[23] Nelson W.J., Traub P. (1983). Proteolysis of Vimentin and Desmin by the Ca^2+^-Activated Proteinase Specific for These Intermediate Filament Proteins. Mol. Cell. Biol..

[24] Sharma S., Mücke N., Katus H.A., Herrmann H., Bär H. (2009). Disease mutations in the “head” domain of the extra-sarcomeric protein desmin distinctly alter its assembly and network-forming properties. J. Mol. Med. Berl. Ger..

[25] Pica E.C., Kathirvel P., Pramono Z.A.D., Lai P.-S., Yee W.-C. (2008). Characterization of a novel S13F desmin mutation associated with desmin myopathy and heart block in a Chinese family. Neuromuscul. Disord..

[26] van Tintelen J.P., Van Gelder I.C., Asimaki A., Suurmeijer A.J.H., Wiesfeld A.C.P., Jongbloed J.D.H., Wijngaard A.v.D., Kuks J.B., van Spaendonck-Zwarts K.Y., Notermans N. (2009). Severe cardiac phenotype with right ventricular predominance in a large cohort of patients with a single missense mutation in the DES gene. Heart Rhythm.

[27] Arbustini E., Pasotti M., Pilotto A., Pellegrini C., Grasso M., Previtali S., Repetto A., Bellini O., Azan G., Scaffino M. (2006). Desmin accumulation restrictive cardiomyopathy and atrioventricular block associated with desmin gene defects. Eur. J. Heart Fail..

[28] Kouloumenta A., Mavroidis M., Capetanaki Y. (2007). Proper perinuclear localization of the TRIM-like protein myospryn requires its binding partner desmin. J. Biol. Chem..

[29] Diokmetzidou A., Tsikitis M., Nikouli S., Kloukina I., Tsoupri E., Papathanasiou S., Mavroidis M., Capetanaki Y. (2016). Strategies to Study Desmin in Cardiac Muscle and Culture Systems. Methods Enzymol..

[30] Pierce B.G., Hourai Y., Weng Z. (2011). Accelerating Protein Docking in ZDOCK Using an Advanced 3D Convolution Library. PLoS ONE.

[31] Hess B., van der Vegt N.F.A. (2006). Hydration thermodynamic properties of amino acid analogues: A systematic comparison of biomolecular force fields and water models. J. Phys. Chem. B.

[32] Sellis D., Vlachakis D., Vlassi M. (2009). Gromita: A Fully Integrated Graphical User Interface to Gromacs 4. Bioinforma. Biol. Insights.

[33] Guex N., Peitsch M.C. (1997). SWISS-MODEL and the Swiss-PdbViewer: An environment for comparative protein modeling. Electrophoresis.

[34] Laskowski R.A., Rullmannn J.A., MacArthur M.W., Kaptein R., Thornton J.M. (1996). AQUA and PROCHECK-NMR: Programs for checking the quality of protein structures solved by NMR. J. Biomol. NMR.

[35] Bowie J.U., Lüthy R., Eisenberg D. (1991). A method to identify protein sequences that fold into a known three-dimensional structure. Science.

[36] Pettersen E.F., Goddard T.D., Huang C.C., Couch G.S., Greenblatt D.M., Meng E.C., Ferrin T.E. (2004). UCSF Chimera—A visualization system for exploratory research and analysis. J. Comput. Chem..

[37] Lefrancois S., Zeng J., Hassan A.J., Canuel M., Morales C.R. (2003). The lysosomal trafficking of sphingolipid activator proteins (SAPs) is mediated by sortilin. EMBO J..

[38] Jumper J., Evans R., Pritzel A., Green T., Figurnov M., Ronneberger O., Tunyasuvunakool K., Bates R., Žídek A., Potapenko A. (2021). Highly accurate protein structure prediction with AlphaFold. Nature.

[39] Varadi M., Anyango S., Deshpande M., Nair S., Natassia C., Yordanova G., Yuan D., Stroe O., Wood G., Laydon A. (2022). AlphaFold Protein Structure Database: Massively expanding the structural coverage of protein-sequence space with high-accuracy models. Nucleic Acids Res..

[40] Mimaki M., Wang X., McKenzie M., Thorburn D.R., Ryan M.T. (2012). Understanding mitochondrial complex I assembly in health and disease. Biochim. Biophys. Acta BBA-Bioenerg..

[41] Diokmetzidou A., Soumaka E., Kloukina I., Tsikitis M., Makridakis M., Varela A., Davos C.H., Georgopoulos S., Anesti V., Vlahou A. (2016). Desmin and αB-crystallin interplay in maintenance of mitochondrial homeostasis and cardiomyocyte survival. J. Cell Sci..

[42] Dayal A.A., Medvedeva N.V., Nekrasova T.M., Duhalin S.D., Surin A.K., Minin A.A. (2020). Desmin Interacts Directly with Mitochondria. Int. J. Mol. Sci..

[43] Milner D.J., Mavroidis M., Weisleder N., Capetanaki Y. (2000). Desmin Cytoskeleton Linked to Muscle Mitochondrial Distribution and Respiratory Function. J. Cell Biol..

[44] Capetanaki Y., Bloch R.J., Kouloumenta A., Mavroidis M., Psarras S. (2007). Muscle intermediate filaments and their links to membranes and membranous organelles. Exp. Cell Res..

[45] Capetanaki Y., Papathanasiou S., Diokmetzidou A., Vatsellas G., Tsikitis M. (2015). Desmin related disease: A matter of cell survival failure. Curr. Opin. Cell Biol..

[46] Tsikitis M., Galata Z., Mavroidis M., Psarras S., Capetanaki Y. (2018). Intermediate filaments in cardiomyopathy. Biophys. Rev..

[47] Tuppen H.A.L., Hogan V.E., He L., Blakely E.L., Worgan L., Al-Dosary M., Saretzki G., Alston C.L., Morris A.A., Clarke M. (2010). The p.M292T NDUFS2 mutation causes complex I-deficient Leigh syndrome in multiple families. Brain J. Neurol..

[48] Loeffen J., Elpeleg O., Smeitink J., Smeets R., Stöckler-Ipsiroglu S., Mandel H., Sengers R., Trijbels F., van den Heuvel L. (2001). Mutations in the complex I NDUFS2 gene of patients with cardiomyopathy and encephalomyopathy. Ann. Neurol..

[49] Dunham-Snary K.J., Wu D., Potus F., Sykes E.A., Mewburn J.D., Charles R.L., Eaton P., Sultanian R.A., Archer S.L. (2019). Ndufs2, a Core Subunit of Mitochondrial Complex I, Is Essential for Acute Oxygen-Sensing and Hypoxic Pulmonary Vasoconstriction. Circ. Res..

[50] Liu L., Qi L., Knifley T., Piecoro D.W., Rychahou P., Liu J., Mitov M.I., Martin J., Wang C., Wu J. (2019). S100A4 alters metabolism and promotes invasion of lung cancer cells by up-regulating mitochondrial complex I protein NDUFS2. J. Biol. Chem..

[51] Jain S., Hu C., Kluza J., Ke W., Tian G., Giurgiu M., Bleilevens A., Campos A.R., Charbono A., Stickeler E. (2022). Metabolic targeting of cancer by a ubiquinone uncompetitive inhibitor of mitochondrial complex I. Cell Chem. Biol..

[52] Timón-Gómez A., Nývltová E., Abriata L.A., Vila A.J., Hosler J., Barrientos A. (2018). Mitochondrial Cytochrome c Oxidase Biogenesis: Recent Developments. Semin. Cell Dev. Biol..

[53] Brischigliaro M., Zeviani M. (2021). Cytochrome *c* oxidase deficiency. Biochim. Biophys. Acta BBA-Bioenerg..

[54] Yoshida A., Rzhetsky A., Hsu L.C., Chang C. (1998). Human aldehyde dehydrogenase gene family. Eur. J. Biochem..

[55] Lorenzo C., Delgado P., Busse C.E., Sanz-Bravo A., Martos-Folgado I., Bonzon-Kulichenko E., Ferrarini A., Gonzalez-Valdes I.B., Mur S.M., Roldán-Montero R. (2021). ALDH4A1 is an atherosclerosis auto-antigen targeted by protective antibodies. Nature.

[56] Schwarz N., Leube R.E. (2016). Intermediate Filaments as Organizers of Cellular Space: How They Affect Mitochondrial Structure and Function. Cells.

[57] Matsuda J., Kido M., Tadano-Aritomi K., Ishizuka I., Tominaga K., Toida K., Takeda E., Suzuki K., Kuroda Y. (2004). Mutation in saposin D domain of sphingolipid activator protein gene causes urinary system defects and cerebellar Purkinje cell degeneration with accumulation of hydroxy fatty acid-containing ceramide in mouse. Hum. Mol. Genet..

[58] Fujita N., Suzuki K., Vanier M.T., Popko B., Maeda N., Klein A., Henseler M., Sandhoff K., Nakayasu H., Suzuki K. (1996). Targeted disruption of the mouse sphingolipid activator protein gene: A complex phenotype, including severe leukodystrophy and wide-spread storage of multiple sphingolipids. Hum. Mol. Genet..

[59] Oji Y., Hatano T., Ueno S.-I., Funayama M., Ishikawa K., Okuzumi A., Noda S., Sato S., Satake W., Toda T. (2020). Variants in saposin D domain of prosaposin gene linked to Parkinson’s disease. Brain.

[60] Koochekpour S., Lee T.-J., Sun Y., Hu S., Grabowski G.A., Liu Z., Garay J. (2008). Prosaposin is an AR-target gene and its neurotrophic domain upregulates AR expression and activity in prostate stromal cells. J. Cell. Biochem..

[61] Wu Y., Sun L., Zou W., Xu J., Liu H., Wang W., Yun X., Gu J. (2012). Prosaposin, a regulator of estrogen receptor alpha, promotes breast cancer growth. Cancer Sci..

[62] Jiang Y., Zhou J., Luo P., Gao H., Ma Y., Chen Y.-S., Li L., Zou D., Zhang Y., Jing Z. (2018). Prosaposin promotes the proliferation and tumorigenesis of glioma through toll-like receptor 4 (TLR4)-mediated NF-κB signaling pathway. EBioMedicine.

[63] Lefrancois S., May T., Knight C., Bourbeau D., Morales C.R. (2002). The lysosomal transport of prosaposin requires the conditional interaction of its highly conserved d domain with sphingomyelin. J. Biol. Chem..

[64] Benson M.A., Tinsley C.L., Blake D.J. (2004). Myospryn is a novel binding partner for dysbindin in muscle. J. Biol. Chem..

[65] Benson M.A., Newey S.E., Martin-Rendon E., Hawkes R., Blake D.J. (2001). Dysbindin, a novel coiled-coil-containing protein that interacts with the dystrobrevins in muscle and brain. J. Biol. Chem..

[66] Reynolds J.G., McCalmon S.A., Donaghey J.A., Naya F.J. (2008). Deregulated Protein Kinase A Signaling and Myospryn Expression in Muscular Dystrophy. J. Biol. Chem..

[67] Tsoupri E., Kostavasili I., Kloukina I., Tsikitis M., Miliou D., Vasilaki E., Varela A., Nakos-Bimpos M., Davos C., Mavroidis M. (2021). Myospryn deficiency leads to impaired cardiac structure and function and schizophrenia-associated symptoms. Cell Tissue Res..

[68] Bunk J., Prieto Huarcaya S., Drobny A., Dobert J.P., Walther L., Rose-John S., Arnold P., Zunke F. (2021). Cathepsin D Variants Associated with Neurodegenerative Diseases Show Dysregulated Functionality and Modified α-Synuclein Degradation Properties. Front. Cell Dev. Biol..

[69] Hiraiwa M., Martin B.M., Kishimoto Y., Conner G.E., Tsuji S., O’Brien J.S. (1997). Lysosomal proteolysis of prosaposin, the precursor of saposins (sphingolipid activator proteins): Its mechanism and inhibition by ganglioside. Arch. Biochem. Biophys..

[70] Tayebi N., Lopez G., Do J., Sidransky E. (2020). Pro-cathepsin D, Prosaposin, and Progranulin: Lysosomal Networks in Parkinsonism. Trends Mol. Med..

[71] Scally S.W., Petersen J., Law S.C., Dudek N.L., Nel H.J., Loh K.L., Wijeyewickrema L.C., Eckle S.B., van Heemst J., Pike R.N. (2013). A molecular basis for the association of the HLA-DRB1 locus, citrullination, and rheumatoid arthritis. J. Exp. Med..

[72] Hess J.F., Budamagunta M.S., Aziz A., FitzGerald P.G., Voss J.C. (2013). Electron paramagnetic resonance analysis of the vimentin tail domain reveals points of order in a largely disordered region and conformational adaptation upon filament assembly. Protein Sci..

[73] Guzenko D., Chernyatina A.A., Strelkov S.V., Parry D.A.D., Squire J.M. (2017). Crystallographic Studies of Intermediate Filament Proteins. Fibrous Proteins: Structures and Mechanisms.

[74] Usman S., Aldehlawi H., Nguyen T.K.N., Teh M.-T., Waseem A. (2022). Impact of N-Terminal Tags on De Novo Vimentin Intermediate Filament Assembly. Int. J. Mol. Sci..

[75] Parry D.A., Steinert P.M. (1999). Intermediate filaments: Molecular architecture, assembly, dynamics and polymorphism. Q. Rev. Biophys..

[76] Herrmann H., Aebi U. (2016). Intermediate Filaments: Structure and Assembly. Cold Spring Harb. Perspect. Biol..

[77] Mücke N., Kämmerer L., Winheim S., Kirmse R., Krieger J., Mildenberger M., Baßler J., Hurt E., Goldmann W.H., Aebi U. (2018). Assembly Kinetics of Vimentin Tetramers to Unit-Length Filaments: A Stopped-Flow Study. Biophys. J..

