# Supplementary material for: Unraveling Desmin’s Head Domain Structure and Function
Source: Cells. 2024 Mar 29;13(7):603. doi: 10.3390/cells13070603 (PMC11012097; doi:10.3390/cells13070603)
Supplement: Supplementary file 1 [file cells-13-00603-s001.zip › cells-2840710-supplementary.pdf]

Supplementary Table S1. Desmin head domain binding partners identified by the yeast2-hybrid system. The retest results of co-transformation of each clone in AH109 cells with the bait plasmid pGBKT7-DesminHead or control plasmids are also presented.

<table>
<tr><th rowspan="2">Clone ID</th><th rowspan="2">Clone ID</th><th rowspan="2">Gene information</th><th colspan="2">Retest transformation into pGBKT7-DesminHead</th><th colspan="2">Retest transformation into pGBKT7</th><th colspan="2">Retest transformation into pGBKT7-53</th></tr>
<tr><th>SD/-4</th><th>SD/-4 +X-a-gal</th><th>SD/-4</th><th>SD/-4 + X-a-gal</th><th>SD/-4</th><th>SD/-4 +X-a-gal</th></tr>
<tr><td>1</td><td>H111*</td><td>NM_004550.3| (NADH-coenzyme Q reductase) (NDUFS2)</td><td>50</td><td>+++</td><td></td><td></td><td></td><td></td></tr>
<tr><td>2</td><td>H112*</td><td>NM_005876.5 Striated muscle enriched protein kinase (SPEG)</td><td>20</td><td>+</td><td>-</td><td></td><td></td><td></td></tr>
<tr><td>3</td><td>H115</td><td>NM_001961.4 Eukaryotic translation elongation factor 2 (EEF2</td><td>30</td><td>+++</td><td>2</td><td>-</td><td>2</td><td>-</td></tr>
<tr><td>4</td><td>H124</td><td>NM_001146277.3 Neutral cholesterol ester hydrolase 1 (NCEH1),</td><td>105</td><td>++</td><td>3</td><td>-</td><td>1</td><td>-</td></tr>
<tr><td>5</td><td>H125[&]</td><td>NM_153610.2| cardiomyopathy associated 5 (CMYA5). Synonym: myospryn</td><td>60</td><td>+++</td><td>1</td><td>-</td><td>-</td><td>-</td></tr>
<tr><td>6</td><td>H127</td><td>NM_025163.4 phosphatidylinositol glycan anchor biosynthesis class Z (PIGZ)</td><td>45</td><td>+++</td><td>2</td><td>-</td><td>-</td><td>-</td></tr>
<tr><td>7</td><td>H128</td><td>|NM_153051.1| myotubularin related protein 3 (MTMR3)</td><td>30</td><td>+++</td><td></td><td></td><td></td><td></td></tr>
<tr><td>8</td><td>H130</td><td>XM_054364132.1 multiple PDZ domain crumbs cell polarity complex component (MPDZ), 3</td><td>25</td><td>++</td><td></td><td></td><td></td><td></td></tr>
<tr><td>9</td><td>H131</td><td>gb| AY429111.1| thyroid stimulating hormone receptor mRNA, (TSHR)</td><td>50</td><td>+++</td><td>-</td><td>-</td><td>-</td><td>-</td></tr>
<tr><td>10</td><td>H132*</td><td>NM_003748.2| Aldehyde dehydrogenase 4 family, member A1 (ALDH4A1)</td><td>22</td><td>+++</td><td></td><td></td><td></td><td></td></tr>
<tr><td>11</td><td>H134</td><td>NM_003977.4| aryl hydrocarbon receptor interacting protein (AIP)</td><td>200</td><td>+++</td><td>6</td><td>-</td><td>1</td><td>-</td></tr>
<tr><td>12</td><td>H135*</td><td>NM_199168.4| C-X-C motif chemokine ligand 12 (CXCL12)</td><td>25</td><td>+++</td><td>-</td><td>-</td><td>2</td><td>-</td></tr>
<tr><td>13</td><td>H136*</td><td>GenBank: U59167.1 Human desmin mRNA (DES)</td><td>25</td><td>++</td><td></td><td></td><td></td><td></td></tr>
<tr><td>14</td><td>H139</td><td>NM_000975.5| ribosomal protein L11 (RPL11)</td><td>60</td><td>+++</td><td>-</td><td>-</td><td>-</td><td>-</td></tr>
<tr><td>15</td><td>H140</td><td>NM_014809.2| Homo sapiens KIAA0319 (KIAA0319)</td><td>26</td><td>++</td><td>1</td><td>-</td><td>-</td><td>-</td></tr>
<tr><td>16</td><td>H143</td><td>Haplogroup H11a2a2d mitochondrion, complete genome. Sequence ID: OR807012.1 cytochrome c oxidase subunit I (COXI), protein ID: WPT10523.1</td><td>73</td><td>++</td><td>1</td><td>-</td><td>-</td><td>-</td></tr>
<tr><td>17</td><td>H144</td><td>NM_025233.4| Coenzyme A synthase (COASY)</td><td>6</td><td>+-</td><td>-</td><td>-</td><td>-</td><td>-</td></tr>
<tr><td>18</td><td>H145*</td><td>|NM_002778.1| prosaposin (PSAP)</td><td>65</td><td>+++</td><td>-</td><td>-</td><td>-</td><td>-</td></tr>
<tr><td>19</td><td>H155</td><td>NM_182985.5 tripartite motif containing 69 (TRIM69)</td><td>35</td><td>+++</td><td>-</td><td>-</td><td>-</td><td>-</td></tr>
<tr><td>20</td><td>H158*</td><td>NM_001909.5| cathepsin D (CTSD)</td><td>>300</td><td>+++</td><td>1</td><td>-</td><td>6</td><td>-</td></tr>
<tr><td>21</td><td>H164</td><td>NM_003875.3 guanine monophosphate synthase (GMPS)</td><td>>150</td><td>+++</td><td>-</td><td>-</td><td>-</td><td>-</td></tr>
<tr><td>22</td><td>H165</td><td>NM_020383.4| X-prolyl aminopeptidase 1 (XPNPEP1)</td><td>>300</td><td>+++</td><td>6</td><td>-</td><td>7</td><td>-</td></tr>
</table>

*More than 2 clones; [&] published, see PMID: 17872945.

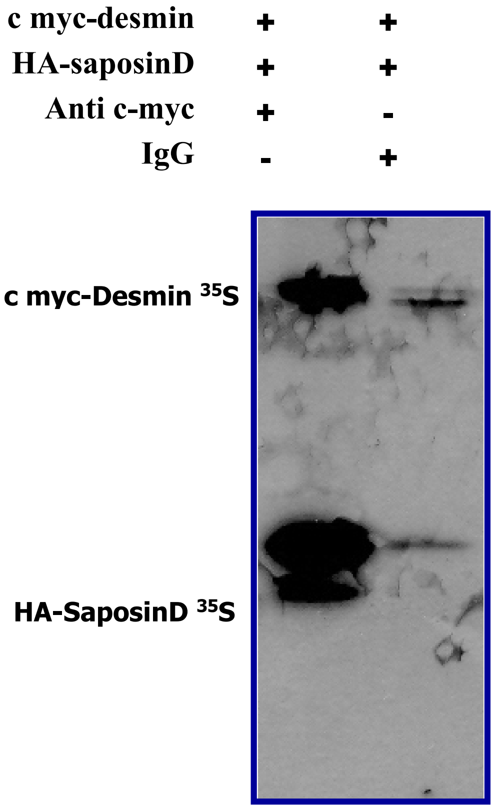

<u>Supplementary Figure S1:</u> Direct interaction of desmin with saposinD. Desmin and saposinD were labelled with [35S] methionine with a TNT Quick Coupled Transscription/Translation System, and were expressed with an epitope tag c-myc and HA respectively. Coimmunoprecipitation analysis shows that c-myc-Desmin interacts with HA-saposin D. (IgG: isotype IgG to anti c-myc antibody, used as negative control)..
